# Physico-chemical behaviour and microbiological suitability of residual smectitic soils mixed with two mineralized waters for therapeutic and dermocosmetic applications

**DOI:** 10.1007/s00484-025-03056-6

**Published:** 2026-01-13

**Authors:** André Valente, Fernando Rocha, Ângela Cunha, Denise Terroso, Cristina Sequeira, Eduardo Ferreira da Silva

**Affiliations:** 1 GeoBioTec Research Centre, Department of Geosciences, University of Aveiro, Campus de Santiago, Aveiro, 3810-193 Portugal; 2 Department of Biology & Centre for Environmental and Marine Studies (CESAM), University of Aveiro, Campus de Santigo, Aveiro, 3810-193 Portugal

**Keywords:** Peloids, Physical chemistry, Microbiology, Therapeutic applications, Dermocosmetic

## Abstract

Peloid is a matured mud with healing and/or cosmetic properties, composed of a complex mixture of mineral or seawater with a clay-based material, that requires quality control prior to its application in therapeutic and dermocosmetic treatments. In this research, physico-chemical and biological analyses were performed to assess influence of the two mineralized waters on three residual smectitic soils. Seawater increased the electrical conductivity values of peloids (from 0.3 to 0.5 mS/cm to 68.0–73.8 mS/cm) and their organic matter content (from 2.6 to 4.7% to around 7%), whereas thermo-mineral water enhanced the cation exchange capacity (from 38.4 to 70.0 meq/100 g to 55.2–86.6 meq/100 g). The pH of peloids remained alkaline, and zeta potential values were stable throughout the maturation period. The concentrations of Pb, Co, Ni and V in samples exceed the acceptable limits established for cosmetic and pharmaceutical products, hence further dermal bioacessibility assessment are required to substantiate their clinical safety before therapeutic use. Moreover, fecal indicator bacteria were not detected in the peloids, however thermo-mineral water peloids showed fungal contents slightly above recommended microbiological limits. The physico-chemical and microbiological characterization suggests that these peloids have potential therapeutic values, although further thermal and rheological characterization are required to assess their suitability.

## Introduction

The use of clays as therapeutic and dermocosmetic agents, particularly smectitic clay, remains a common practice worldwide, whether through the preservation of native cultural traditions, pharmaceutical formulations, or integrative health and well-being practices (Carretero 2020a).

The conventional use of clay-based materials stems from its abundance on the earth’s surface and its well-established therapeutic potential as an anti-inflammatory, antiseptic, bactericidal, analgesic, and detoxifying agent (Gomes et al. 2021).

The external use of clay materials is typically applied in the form of hot clay poultices, known as peloids, or mud/clay baths, in dermatological treatments (psoriasis and acne) (Gomes et al. 2021; Zhang et al. 2023) and in inflammations, namely, arthritis, tendonitis and rheumatism (Kasapoğlu et al., 2017; Bastos and Rocha 2023). Among the various clay-based treatments, the most recognised nowadays is pelotherapy, which consists of treatments based on peloids. In 2012, a multidisciplinary group of researchers, from several domains, who had been working in the pelotherapy field until then, suggested the following definition (Gomes et al. 2013): *a peloid is a maturated mud or muddy suspension (more precisely muddy dispersion) with healing and/or cosmetic properties composed of a complex mixture of fine-grained materials (mineral and/or organic)*,* with mineral water*,* seawater*,* salt-lake water*,* and commonly organic compounds from biological metabolic activity.*

Over the last decades, several studies across the Europe have been conducted, about the influence of linking clays with different mineralized waters with therapeutic properties: Veniale et al. (2004), Tateo et al. (2006; 2010), Gámiz et al. (2009), Carretero et al. (2007; 2010), Karakaya et al. (2010, 2017), Quintela et al. (2015), Pozo et al. (2018), Fernández-González et al. (2021), Bastos and Rocha (2023), Lampropoulou et al. (2023), Vukićević et al. (2024), among others. The maturation period, light, temperature and stirring are the most critical parameters in peloids preparation because it leads to the merging of three interrelated phases: the solid phase represented by clay materials, the liquid phase represented by mineralized water and the biotic phase represented by microflora (Carretero et al. 2007). The influence of mineralized waters on the maturation process, also known as “ageing”, enhances the physico-chemical properties, improve the organic matter content derived from the metabolic activity of microorganisms colonizing the solid-liquid interface, while also improving cation exchange capacity, plasticity and cooling time (Veniale et al. 2004; Tateo et al. 2010; Rebelo et al. 2015). A peloid suitable for therapeutic and dermocosmetic application must have specific characteristics such as low cooling time, high cation exchange capacity, as well as good adhesiveness, easy mashing and low abrasiveness that offers a pleasant sensation when applied to human skin (Gomes et al. 2013).

Natural materials intended for use as peloids should be subject to quality monitoring to determine the presence and concentration of heavy metals, trace elements, and pathogenic microorganisms in order to assure the safety of the user (Mourelle et al. 2021; Bastos and Rocha 2023). Commonly, potentially toxic elements (PTE’s) appear in the chemical composition of raw materials due to their occurrence in nature, but also caused by anthropogenic sources like car traffic, agricultural production, or mining (Gomes and Silva 2007). An excess of PTE’s in the human body - antimony (Sb), arsenic (As), cadmium (Cd), lead (Pb) and/or mercury (Hg) - can cause various forms of cancer (Ebrahimi et al. 2020; Ali et al. 2024). There are some European regulations for quality control of peloids, however, according to Bastos and Rocha (2023), dosage and exposure time are required for safety compliance in thermal treatments and dermocosmetic industries. Dermal bioaccessibility can be defined as the fraction of an element that can be released from the peloid and become available for penetration through skin (Stefaniak et al. 2014). The assessment of dermal bioacessibility provide a reasonable measure of clinical safety of peloids prior to therapeutic application (López-Galindo et al. 2007). Moreover, peloids should be free of pathogenic microorganisms, such as fungi and fecal contaminants, which can pose serious health risks (Quintela et al. 2012; Kataržytė et al. 2024).

The main goal of this research is to assess the influence of the two mineralized waters on the physico-chemical and microbiological characteristics of three residual smectitic soils. For this purpose, the following objectives have been defined: (1) identify the minor and trace elements of samples, (2) to characterize the physico-chemical and microbiological parameters of peloids, (3) to highlight the main changes after maturation process, (4) and to compare the values obtained to previous research and pre-establish parameters in literature.

## Materials and methods

### Materials

In line with previous research conducted by Valente et al. (2025), the samples consist of three residual smectitic soils (Beja, Bena – Benavila, and Monta – Montargil) outcropping in Alentejo region, south of Portugal, resulting from the weathering of magmatic rocks, and two mineralized waters: thermo-mineral water and seawater.

The mineralogical composition of residual soils (Beja, Bena and Monta) is mainly composed of smectite (50–72%). The influence of mineralized waters was quite clear on the mineralogical composition of peloids and, consequently, on their chemical composition. Thermo-mineral water peloids (Beja-T, Bena-T and Monta-T) had a slight increase of smectite (58–76%), whereas seawater peloids (Beja-S, Bena-S and Monta-S) had a decrease (39–54%) duo to the crystallization of newly formed minerals (halite and gypsum) (Table 1). The concentrations of Na_2_O and Cl have substantially increase in seawater peloids to 2.1–3.7% (from 0.1 to 1.2%) and to 7.0–8.8% (from below quantification limit), respectively.

**Table 1:** Mineralogical and chemical composition of samples (adapted from Valente et al. 2025).

Samples	Mineralogical composition													
	**Sme**	**Kln**	**Fsp**		**Fe-ox**	**Carbo**	**Hlt**	**Gpm**		**Qtz**	**Op**	**Ant**	**Zlt**	
Beja	50	1	24		9	-	-	-		7	7	-	2	
Beja-T	58	2	16		8	-	-	-		7	6	-	3	
Beja-S	43	2	11		10	-	26	1		4	3	-	-	
Bena	72	1	1		6	17	3	-		3	-	-	-	
Bena-T	76	-	1		6	14	-	-		3	-	-	-	
Bena-S	54	-	4		7	8	23	1		3	-	-	-	
Monta	57	4	-		7	22	-	-		1	-	7	2	
Monta-T	66	1	1		7	19	-	-		1	-	5	-	
Monta-S	39	2	3		6	20	22	1		2	-	5	-	
Chemical composition														
	**Na**_**2**_**O**	**MgO**		**Al**_**2**_**O**_**3**_	**SiO**_**2**_	**SO**_**3**_	**Cl**	**K**_**2**_**O**	**CaO**			**Fe**_**2**_**O**_**3**_		**Br**
Beja	1.2	4.3		20.1	52.4	bql	bql	0.3	5.2			6.9		-
Beja-T	1.0	4.0		20.5	51.1	bql	0.1	0.3	4.1			7.0		bql
Beja-S	3.7	6.2		15.9	35.8	2.3	8.8	0.8	3.1			4.2		0.1
Bena	0.1	5.2		19.3	52.8	bql	bql	0.7	4.4			3.1		-
Bena-T	0.1	5.0		18.8	52.2	bql	0.1	0.6	4.8			3.0		bql
Bena-S	3.1	7.3		14.2	37.6	1.3	8.5	1.0	3.3			1.8		bql
Monta	0.1	5.9		15.1	48.2	bql	bql	0.3	5.6			11.2		-
Monta-T	0.2	6.6		15.3	48.4	bql	0.1	0.4	10.9			12.2		bql
Monta-S	2.1	8.0		10.6	32.2	1.4	7.0	0.7	6.8			6.2		bql

*Sme *smectite, *Kln* kaolinite, *Fsp *feldspars, *Carbo *carbonates, *Hlt* halite, *Gpm *gypsum, *Qtz *quartz, *Op *opal, *Ant *anatase, *Zlt *zeolite, *bql *below quantification limit, - non-detected

Regarding the chemical composition of waters, thermo-mineral water is sulphurous, hydroxylated and sodium chloride with a highly alkaline pH of 11.6 and electrical conductivity of 0.64 mS/cm. Likewise, seawater is sodium chloride, with sulphate and bicarbonate anions and magnesium, calcium and potassium cations, with an alkaline pH value of 7.9 and an electrical conductivity of 56.7 mS/cm (Valente et al. 2025).

To assess the potential therapeutic value of samples, a chemical and physico-chemical characterization of residual smectitic soils (Beja, Bena and Monta) and respective thermo-mineral waters peloids (Beja-T, Bena-T and Monta) and seawater peloids (Beja-S, Bena-S and Monta-S), were carried out according to Quintela et al. (2012), Rebelo et al. (2015) and Bastos and Rocha (2023). Moreover, the microbiological suitability of peloids was assessed following Baldovin et al. (2020), Bastos and Rocha (2023) and Kataržytė et al. (2024).

## Methods

### Chemical analyses

The minor and trace elements on staring samples and respective peloids under study were analyzed by X-ray fluorescence (XRF), using AXIOS PW4400/40 XRF-Panalytical equipment (manufactured by Marvel Panalytical, Almelo, Netherlands), operating on a Rb tube under argon/methane. This technique allows the determination of the subsequent minor and trace elements concentrations (mg/kg): Br, Ce, Co, Cr, Cs, Cu, Ga, La, Mn, Mo, Nb, Nd, Ni, Rb, Pb, Sc, Sm, Sn, Th, U, V, W, Y, Zn and Zr.

### Physico-chemical analyses

The pH was measured following the (ISO, 10390: 2021) standard, using a calibrated Hanna Instruments model HI 9126 device (manufactured by Hanna Instruments, Limena, Italy), with an accuracy of ± 0.05, in a 1:5 soil-distilled water mixture. Electrical conductivity (EC) was measured using a calibrated Hanna Instruments model HI 9033 Multi Range device (manufactured by Hanna Instruments, Limena, Italy), with an accuracy of ± 0.05, in accordance with the (ISO, 11265:^1994^) standard, in a 1:5 soil-water mixture. The organic matter (OM) was measured according to the procedure proposed by Van Reeuwijk (2002), that consists of burning organic material and relation of weights before and after the burning process. The cation exchange capacity (CEC) was assessed through a process involving saturation, filtration, distillation and titration to determine the exchangeable ions. Primarily, a solution of ammonium acetate (CH_3_COONH_4_) was used for saturation; then, the solution was filtered using Macherey-Nagel MN640d filter paper (manufactured by Macherey-Nagel, Düren, Germany) under vacuum extraction to identify exchangeable cations (Na^+^, K^+^, Mg^2+^ and Ca^2+^). Meanwhile, to recognise the total of exchangeable cations (mg/L) it was used an Agilent Technologies 7700 Series Inductively Couples Plasma Mass Spectrometry (ICP-MS) device (manufactured by Agilent, California, USA). Afterward, the excess of ammonium acetate was removed with ethanol and tested using Nessler’s reagent. During the distillation process, magnesium oxide (MgO) was added, and the material was distilled into a flask containing a 4% boric acid (H_3_BO_3_) solution and the bromocresol indicator (0.1%). Lastly, the sample was titrated with 0.1 N hydrochloric acid (HCl), following the procedure outlined by Gomes (1988). The zeta potential (ZP) is determined using Malvern’s ZetaSizer Nano ZS equipment, model Zen 3500 (manufactured by Malvern Panalytical, Malvern, United Kingdom). The supernatant was introduced into folded 1 mL capacity capillary zeta potential (DTS1061, Malvern Instruments) and the measurements were performed in triplicate at a temperature of 25 °C and 45 °C, and, for each replicate, 20 data points were collected.

The maturation process is described in Valente et al. (2025), where the three residual smectitic soils resulted in the following six peloids: Beja-T, Bena-T, Monta-T (matured with thermo-mineral water), Beja-S, Bena-S and Monta-S (matured with seawater).

### Biological analyses

The microbiological characterization of the peloids was performed using culture-dependent techniques based on colony counts. Prior to inoculation, 1:10 w/v (10^− 1^) solid–liquid suspensions of the peloid were prepared in sterile Ringer solution to ensure adequate homogenization on the respective culture media. The suspension was vigorously vortexed and used for a further decimal dilution (10^− 2^) in Ringer solution.

The concentration of total mesophilic bacteria was determined by the pour plating method (ISO 6222:^1999^) in Plate Count Agar (manufactured by Liofilchem, Roseto degli Abruzzi, Italy) of triplicate aliquots of each dilution (10^− 1^ and 10^− 2^). Cultures were incubated for 48 h, at 37 °C. The counts in the replicates of the most suitable dilution were averaged, corrected for the dilution factor and expressed as Colony Forming Units per gram of peloid (CFU g^−1^).

Total coliforms and *E. coli* were quantified by the pour plating method (ISO 9308-1:^2014^) in Chromocult Coliform Agar (manufactured by Merck, Darmstadt, Germany). Triplicate plates of the 10^− 1^ dilution were prepared. Cultures were incubated aerobically at 37 °C for 24 h. Following incubation, cultures were observed for the development of colonies with characteristic colours. Dark blue to violet colonies were counted as *E. coli*. The counts of each replicate set were averaged, corrected for the dilution factor and expressed as CFU g⁻¹.

Enterococci were quantified by the pour plating method (ISO 7899-2:^2000^) in KF Streptococcus Agar (manufactured by Merck, Darmstadt, Germany) supplemented with 0.01% TTC (2, 3, 5-triphenyl tetrazolium chloride). Triplicate plates of the 10⁻¹ dilution were prepared. Cultures were incubated aerobically at 37 °C for 48 h. Following incubation, cultures were observed for the development of colonies with characteristic colours. Red colonies were counted as enterococci. The counts of each replicate set were averaged, corrected for the dilution factor, and expressed as CFU g⁻¹.

The determination of yeasts and moulds (ISO 21527:^2008^) was carried out by spreading 200 µl aliquots of each dilution onto Dichloran-Rose Bengal Chloramphenicol Agar (manufactured by Liofilchem, Roseto degli Abruzzi, Italy). For each dilution, five replicate plates were prepared. The inoculated plates were incubated aerobically at 25 °C for five days. Following incubation, colonies of yeasts and moulds were enumerated in the most suitable dilution, corrected for the dilution factor and the aliquot volume, and averaged. The results were expressed as CFU g^−1^.

In parallel with the culture-dependent quantification of microorganisms, enrichment cultures of each peloid were prepared by inoculating 1 g of material into 100 mL of peptone-buffered solution (manufactured by Merck, Darmstadt, Germany). Cultures were incubated at 37 °C for 48 h. Aliquots of the enrichment cultures were spread-plated onto Chromocult Coliform Agar and KF Streptococcus Agar supplemented with 0.01% TTC to confirm the absence of *E. coli* and enterococci in 1 g of the peloid sample.

## Results

### Minor and trace elements

The Table 2 reveals the concentration (mg/kg) of minor and trace elements for the residuals smectitic soils (Beja, Bena and Monta) and the following peloids matured with thermo-mineral water (Beja-T, Bena-T and Monta-T) and seawater (Beja-S, Bena-S and Monta-S).

**Table 2:** Concentration (mg/kg) of minor and trace elements of starting samples and following peloids

Class		USP/ICH	Beja	Beja-T	Beja-S	Bena	Bena-T	Bena-S	Monta	Monta-T	Monta-S
1	**Pb**	**5**	33.0	32.4	20.8	10.7	13.3	6.5	5.9	9.1	3.9
2 A	**Co**	**5**	46	140	90	-	-	-	24	20	20
	**Ni**	**20**	220	210	140	30	30	40	70	80	40
	**V**	**10**	110	80	60	40	30	20	180	210	110
3	**Cr**	**1100**	620	550	390	60	60	40	280	260	160
	**Cu**	**300**	100	220	90	30	40	30	90	180	100
	**Mo**	**1500**	1.1	0.8	0.9	1.3	1.0	-	1.0	1.2	0.9
Other Elements	**Sn**		3.5	8.0	5.1	4.7	4.9	4.0	3.1	6.2	3.5
	**Br**		3.9	7.0	aql	-	4.3	aql	-	3.5	aql
	**Ce**		23	20	10	70	70	40	-	-	-
	**Cs**		-	5.8	-	-	-	-	5.7	-	7.1
	**Ga**		12.0	12.0	8.4	14.8	14.6	9.8	10.7	9.5	6.7
	**La**		-	-	-	34.2	28.5	16.1	-	-	-
	**Mn**		1680	1150	920	490	590	390	820	1140	760
	**Nb**		1.5	1.9	0.9	8.0	10.1	6.7	1.1	1.3	-
	**Nd**		8.8	7.3	6.3	23.8	22.6	15.1	-	6.4	-
	**Rb**		19.2	19.0	18.3	30.0	34.1	28.1	9.5	11.4	13.2
	**Sc**		25.4	26.1	21.9	16.5	15.4	11.3	30.4	35.9	23.7
	**Sm**		-	7.1	-	-	-	-	-	-	-
	**Th**		3.0	2.8	-	10.1	10.0	7.9	-	-	-
	**U**		-	-	-	-	1.3	-	-	1.9	1.8
	**W**		-	3.9	-	6.0	6.9	4.8	-	-	-
	**Y**		10.1	9.2	5.7	9.1	12.4	7.3	4.8	8.8	5.7
	**Zn**		70	90	40	30	40	10	70	100	40
	**Zr**		50.0	47.2	28.6	20.6	32.3	18.9	12.7	14.4	8.0

Non-detected; *aql* above quantification limit, *USP* United States Pharmacopoeia, *ICH* International Conference on Harmonisation

The most representative elements in starting samples (< 63 μm) are chromium (Cr), manganese (Mn) and nickel (Ni) with concentrations between 60 mg/kg (Bena) and 620 mg/kg (Beja), 490 mg/kg (Bena) and 1680 mg/kg (Beja), 30 mg/kg (Bena) and 220 mg/kg (Beja) (Table 2). On one hand, the peloids matured with thermo-mineral water underwent a slightly enrichment in copper (Cu) to 220 mg/kg in Beja-T (from 100 mg/kg in Beja), 40 mg/kg in Bena-T (from 30 mg/kg in Bena) and 180 mg/kg in Monta-T (from 90 mg/kg in Monta), but also in bromine (Br), between 3.5 (Monta-T) and 7.0 mg/kg (Beja-T) and in tin (Sn) between 4.9 (Bena-T) and 8.0 mg/kg (Beja-T). Whereas the concentration of most representative elements generally remained the same compared to the starting samples (Table 2). One the other hand, the peloids matured with seawater underwent a particular enrichment in Br from 3.9 mg/kg in Beja and non-detected in Bena and Monta to higher quantities (around 0.05%), as seen in previous research conducted by Valente et al. (2025). Besides that, the concentrations of most elements decreased. Concerning the most representative elements, concentrations generally decreased. While numerous elements revealed alterations in their concentrations, Cr, Cu and Mn, above all, underwent more pronounced variations during the maturation process. Bastos and Rocha (2023) highlighted the same variations with Cu and Zn, suggesting their possible impact in the transformation of residual soils into peloids.

### Physico-chemical properties

The pH (25 °C) value of starting samples (< 63 μm) is between 8.3 (Beja) and 9.2 (Monta), which is in line with Pereira (1993) and Dias et al. (2004), who obtained similarly values in same study area. Hence, the residual smectitic soils from Alentejo, are considered alkaline and/or very alkaline pH, which is usual for soil samples rich in smectite (Luo et al. 2015). After ageing the starting samples, the thermo-mineral water peloids and seawater peloids showed the following pH (25 °C) values, respectively: 7.9 in Beja-T and 7.1 in Beja-S (from 8.3 in Beja), 8.3 in Bena-T and 7.9 in Bena-S (from 8.9 in Bena) and in 8.5 in Monta-T and 7.9 in Monta-S (from 9.2 in Monta) (Table 3). Veniale et al. (2004) also witnessed that peloids matured with salty water have lower pH than peloids matured with sulphureous water.

**Table 3:** pH, electrical conductivity (mS/cm), organic matter (%) and cation exchange capacity (meq/100 g) of samples

	Beja	Beja-T	Beja-S	Bena		Bena-T	Bena-S	Monta	Monta-T	Monta-S
pH (25 °C)	8.3	7.9	7.1	8.9	8.3		7.9	9.2	8.5	7.9
EC (24.5 °C)	0.4	0.9	73.8	0.5		0.8	70.7	0.3	0.7	68.0
OM	4.7	4.6	7.4	3.5	3.4		7.1	2.6	2.6	7.0
CEC	38.4	55.2	31.4	70.0	86.6		53.6	43.6	57.2	34.6

The EC (24.5 °C) of starting samples (< 63 μm) ranges from 0.3 mS/cm (Monta) to 0.8 mS/cm (Bena) (Table 3). With regard to the peloids obtained after the maturation process, there was an increase in EC values compared to the starting samples, particularly in peloids matured with seawater. In thermo-mineral water peloids, the results slightly increased to between 0.7 mS/cm (Monta-T) and 0.9 mS/cm (Beja-T) (from 0.3 to 0.5 mS/cm), whereas in seawater peloids, the results substantially increased to between 68.0 mS/cm (Monta-S) and 73.8 mS/cm (Beja-S) (Table 3). Bearing in mind that EC is related with dissolved salts and ions, would be expected to seawater pleoids have higher values than the remaining ones (Karakaya and Karakaya 2018). The electrical conductivity of starting samples and peloids are within the range of values reported by Valente et al. (2024) and Özay et al. (2020), respectively.

The OM content of peloids matured with thermo-mineral water remained very similar to the content of starting samples: 4.6% in Beja-T (from 4.7% in Beja), 3.4% in Beja-S (from 3.5% in Bena) and 2.6% in Monta-T (from 2.6% in Monta). However, the content quite growth in Beja-S, Bena-S and Monta-S: 7.4%, 7.1% and 7.0%, respectively (Table 3). The percentages of OM are in line with the loss on ignition at 1000 °C, already determined in the previous study conducted by Valente et al. (2025). Both percentages were mainly related to carbonate content and certain volatile non-carbon components such as gypsum, sulphide minerals, and dehydration of metallic oxyhydroxides (Santisteban et al. 2004).

The CEC values of starting samples range from 38.4 meq/100 g (Beja) to 70.0 meq/100 g (Bena), which is in line with Rebelo et al. (2010), who obtained between 20 and 73 meq/100 g in samples of Alentejo region. The highest CEC value occurred in the sample with greatest smectite concentration (70% in Bena) and lowest value in the sample with smallest concentration (50% in Beja), which is related to the specific surface area of this clay mineral. Moreover, these results are similar to other healing clays with the same application purposes (Cerqueira et al. 2019). Thermo-mineral water contributed to the samples ability to exchange cations as the values increased compared to the starting samples: 55.2 meq/100 g in Beja-T (from 38.4 meq/100 g in Beja), 86.6 meq/100 g in Bena-T (from 70.0 meq/100 g in Bena) and 57.2 meq/100 g in Monta-T (from 43.6 meq/100 g in Monta). Fernández- González et al. (2021) also reported that CEC values slightly increased in matured peloids with thermo-mineral water. Conversely, seawater had a negative influence on the cation exchange capacity of peloids compared to the starting samples: 31.4 meq/100 g in Beja-S, 53.6 meq/100 g in Bena-S and 34.6 meq/100 g in Monta-S (Table 3). Veniale et al. (2004) also noticed that peloids matured with salty waters had lower CEC values and concluded that such negative influence is probably to be referred to agglomeration of fine particles fraction or salts precipitation that stops some surface (re)active sites. Although, the CEC values of peloids from this research are similar than those reported in Quintela et al. (2012), Karakaya et al. (2017) and Fernández-González et al. (2021), which was 10–120 meq/100 g, 10–36 meq/100 g and 10–11 meq/100 g, respectively.

**Fig. 1:**
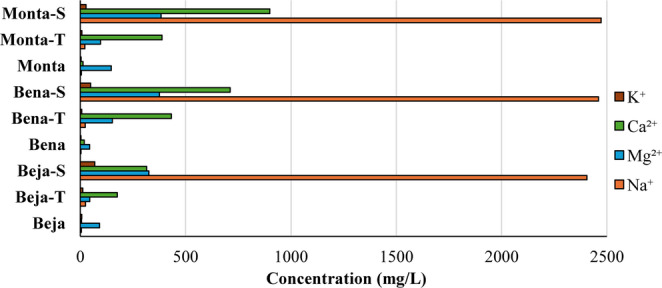
Concentration (mg/L) of exchangeable cations of all samples

The main exchangeable cation in starting samples is Mg^2+^ with a concentration ranging from 44.4 mg/L (Bena) to 146.8 mg/L (Monta), followed by Ca^2+^ (5.8–19.0 mg/L), K^+^ (3.3–7.0 mg/L) and Na^+^ (2.5–5.1 mg/L) (Fig. 1). Over the course of maturation process, the concentration of exchangeable cations in thermo-mineral waters and seawater peloids followed a very dissimilar path, related to the ionic composition of the two different mineralized waters. The main exchangeable cation of Beja-T, Bena-T and Monta-T became Ca^2+^, followed by Mg^2+^, Na^+^ and K^+^. The concentrations vary between 175 and 432 mg/L in Ca^2+^ (from 5.8 to 19.0 mg/L), 44.6–152 mg/L in Mg^2+^ (from 44.4 to 146.8 mg/L), 21.5–24.6 in Na^+^ (from 2.5 to 5.1 mg/L) and 6.5–11.3 mg/L (from 3.3 to 7.0 mg/L). Regarding Beja-S, Bena-S and Monta-S, Na^+^ became the main exchangeable cation, followed by Ca^2+^, Mg^2+^ and K^+^. The concentration ranges from 2405 to 2472 mg/L in Na^+^, 315 to 900 mg/L in Ca^2+^, 326 to 383 mg/L in Mg^2+^ and 26.9 to 68.5 mg/L in K^+^. The seawater ionic composition is dominated by Na^+^, which leads to a strong saturation of this cation in smectite interlayer space. Even though the smectitic clay doesn’t have a high predilection for Na^+^, the overwhelming sodium concentration in water causes it to control the interlayer ion substitution (Meunier 2005; Tateo et al. 2010). Previous research conducted by Valente et al. (2025) had already concluded that Na^+^ was incorporated as an exchange cation through the determination of the smectite crystallochemical formula.

**Fig. 2:**
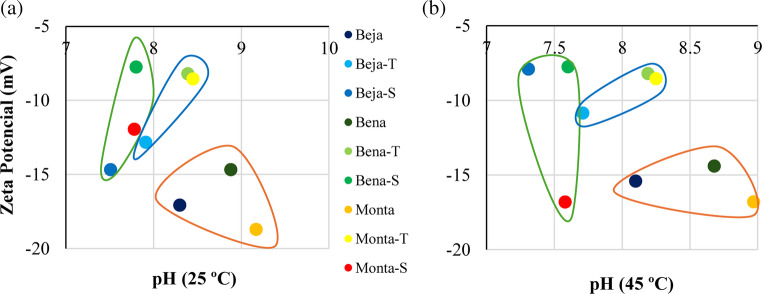
Zeta potential values following different temperatures: (**a**) 25 °C and (**b**) 45 °C. Starting samples surrounded by orange, thermo-mineral water peloids by blue and seawater by green

Over the maturation period, repulsive forces were observed, and even though ZP values are negative for the whole pH interval, it is possible to highlight differences between the starting samples and respective peloids (Fig. 2). At temperature of 25 °C (Fig. 2a) and 45 °C (Fig. 2b), starting samples show slightly more negative zeta potential values (−14.4 - −18.7 mV) than peloids (−7.7 - −14.7 mV), exceptionally sample Monta-S, which at 45 °C display a similar value to Beja, Bena and Monta. The data obtained are in line with Şans et al. (2017) and Bastos and Rocha (2023), which attained values that ranged from − 9.5 to −17.4 mV and from − 10 to −16 mV, respectively.

### Biological characterization

**Fig. 3:**
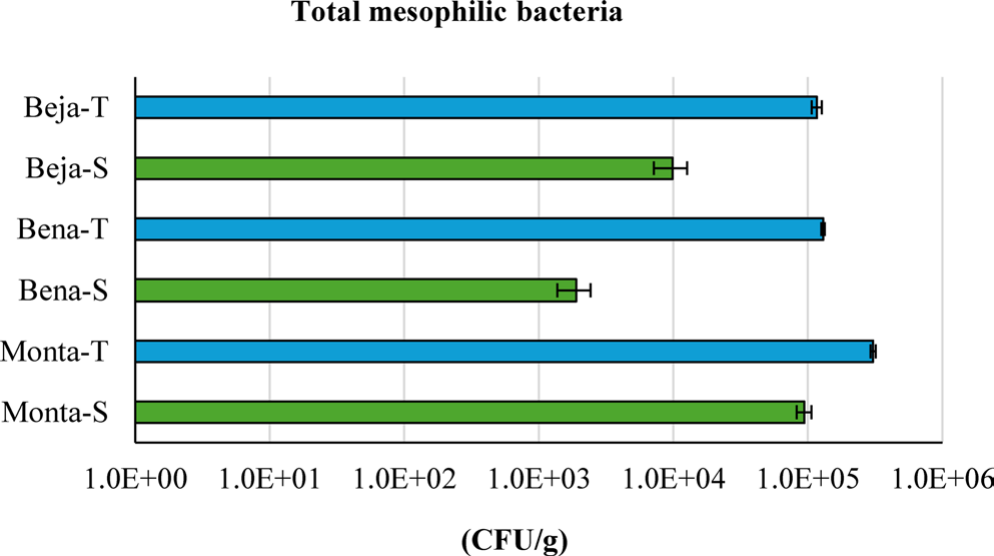
Concentration of total mesophilic bacteria in peloids (thermo-mineral water peloids in blue and seawater peloids in green) after 120 days of maturation. Error bars correspond to the standard deviation of 3 analytical replicates

The concentration of total mesophilic bacteria varied in the range of 10^3^ − 10^5^ CFU/g (Fig. 3). Thermo-mineral water peloids (Beja-T, Bena-T and Monta-T) showed a higher colony-forming units per g (around 10^5^) than seawater peloids (Beja-S, Bena-S and Monta-S) (10^3^ − 10^4^).

Total coliforms and *E. coli* were not detected in the peloids nor in the enrichment cultures, indicating a concentration below 1 CFU/g. Similarly, Enterococci were absent from all the peloid samples and corresponding enrichment cultures, apart from Monta-T peloid, which exhibits an atypical high concentration (5.5 × 10^2^ UFC/g) of these indicators.

**Fig. 4:**
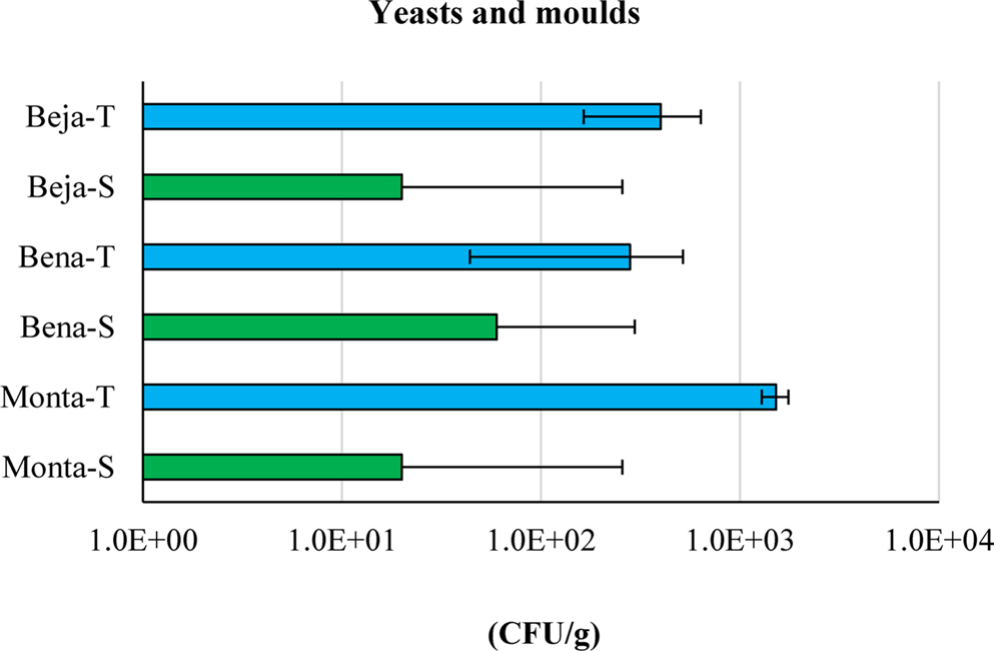
Concentration of fungi (yeasts and moulds) in peloids (thermo-mineral water peloids in blue and seawater peloids in green) after 120 days of maturation. Error bars correspond to the standard deviation of 3 analytical replicates

In this work, a non-specific quantification of yeasts and moulds was performed, assuming an indicator value of this parameter. Fungi were detected in all the samples, in concentrations ranging between 10^1^ and 10^3^ CFU/g (Fig. 4).

## Discussion

Currently, there is a lack of guidelines and regulations that ensures the quality, purity and safety of peloids but, considering that these materials have similarities to cutaneous and transcutaneous drug products, this research was based on the ensuing documentation: USP 40-NF 35 (2017) and ICH Q3D (R2) (2022). According to these guidelines, antimony (Sb), arsenic (As), cadmium (Cd), lead (Pb) and mercury (Hg) are classified as “Class 1” in terms of toxicity level, which means that these elements are human toxicants that have limited or no use in the manufacture of pharmaceuticals bearing in mind that the dermal exposure is expected to be the most important administration route, as the absorption and storage in human body incite the development of several types of cancer (Ebrahimi et al. 2020; Gomes et al. 2021). Moreover, these elemental impurities are strictly forbidden by European Commission - EC No. 1223/2009 (2009). Following the same guidelines, Co, Ni and V are classified as “Class 2A”, since they have relatively high probability of occurrence in the drug product, and Cr, Cu and Mo are classified as “Class 3”, even though they have a reduced probability of occurrence in the drug product related to their low abundance and low potential to be co-isolated with other materials.

In line with the aforementioned guidelines, it’s recommended that elemental impurities do not exceed the following acceptable limits (Table 2): 5 mg/kg in Pb, 5 mg/kg in Co, 20 mg/kg in Ni, 10 mg/kg in V, 1100 mg/kg in Cr, 300 mg/kg in Cu and 1500 mg/kg in Mo. Acceptable limits for the remained elements have not been established due to their low inherent toxicity and/or differences in regional regulations are not addressed in these guidelines. The concentration of Pb (3.9–33.0 mg/kg), Ni (30–220 mg/kg) and V (20–210 mg/kg) exceed the acceptable limits in all samples. Regarding Co, is above the acceptable limit in Beja-clay (Beja, Beja-T and Beja-S) and Monta-clay based material (Monta, Monta-T and Monta-S), with exception in Bena-clay based material (Bena, Bena-T and Bena-S) which is not detected. Despite concentrations of Cr are in accordance with the acceptable limits, it also part of the list by EC (2009), as well as Pb. Lastly, Cu and Mo are the only elements that are below the acceptable limits by the guidelines in all samples (Table 2). Rebelo et al. (2010) also indicate the presence of PTE’s in Alentejo residual soils, with an average concentration of 150 mg/kg in Cr, 75 mg/kg in Cu, 25 mg/kg in Ni, 10 mg/kg in Pb and 100 mg/kg in V.

Although peloids exhibited Pb, Co, Ni and V concentrations above the acceptable limits for cosmetic and pharmaceutical products, these values do not necessarily suggest an immediate restriction of their therapeutic use. Previous study conducted by Wang et al. (2022) shows that total PTE’s concentrations do not correspond to the amount of each element that can be released from the material. In accordance with Stefaniak et al. (2014), the toxicological relevance of these elements depends primarily on their dermal bioacessibility, which determine the fraction effectively release from the peloid matrix and capable of permeating the skin under real application conditions. Tests on synthetic sweat formulations (Bastos et al. 2023; Villegas et al., 2023) and in vitro studies (Chen et al. 2020; Bastos et al. 2024) are essential to support regulatory decisions, demonstrate clinical safety, and establish conditions for controlled use, even when some elements are above the regulatory reference limits. For those reasons, Pb, Co, Ni and V concentrations identified in this study should be interpreted as a requirement for subsequent safety assessment rather than as a prohibitive factor.

There is no endorsed pH value for therapeutic use of peloids, although values between 4 and 8 are the most advisable in order to avoid probable skin irritations (Glavaš et al. 2017) and, according to a study conducted by Quintela et al. (2012), healing clays applied for pharmaceutical and cosmetic purposes has a pH value higher than 6. Considering the alkaline pH values of the starting samples (8.3–9.2) and mineralized waters (11.6 in thermo-mineral water and 7.9 in seawater), it was expected that all peloids would have also an alkaline pH: 7.9–8.5 in thermo-mineral water peloids and 7.1–7.9 in seawater peloids. People with skin illnesses have low pH values, so it is crucial to apply an alkaline product to stabilize the skin’s levels (Proksch 2018). Therefore, the peloids from this research are also within the values measured in diverse peloids for therapeutic and dermoscosmetic applications, for instance, in Turkey (Özay et al. 2020) and Montenegro (Bigovic et al. 2020) and in Brazil (Silva et al. 2015), respectively.

**Fig. 5:**
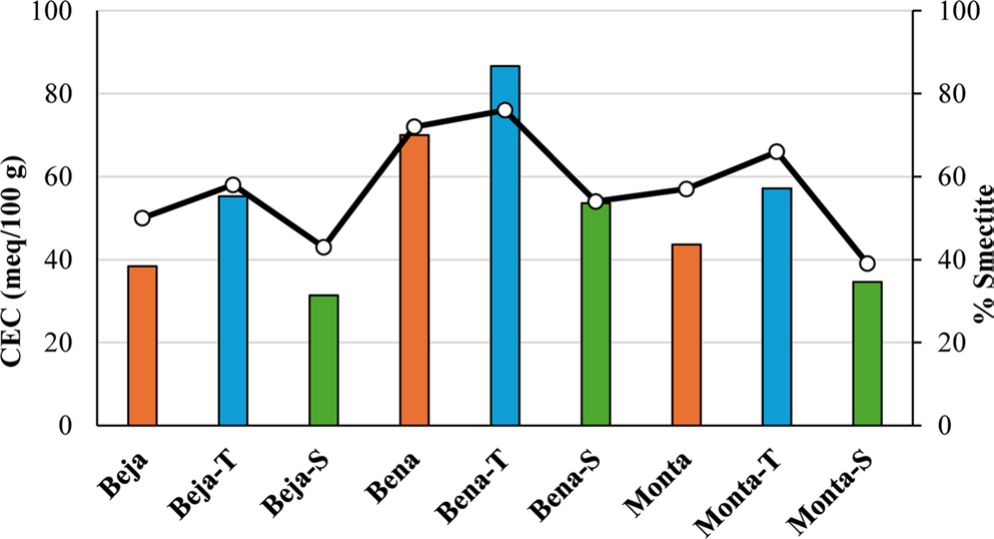
Cation exchange capacity (meq/100 g) of samples

Regarding the cation exchange capacity, the mineralized waters offered different qualities to peloids. The growth of fine particles fraction, particularly the clay fraction rich in smectite (Table 1), played a crucial role in CEC raising values of thermo-mineral water peloids. In literature, smectite are recognized as the clay mineral with the highest cation exchange capacity due to their tendency towards intracrystalline expandability phenomena (Gomes 1988). There is a very strong positive correlation between the smectite content of samples and CEC values (*r* = 0.94), i.e., the higher the content of smectite, the higher the material’s CEC tends to be (Fig. 5). This aspect emphasis the idea that preserving or increasing smectite fraction during maturation is a key factor in the peloids performance.

Peloids with high CEC, which in this case are Beja-T, Bena-T and Monta-T, provide some vital chemical elements for the human body (sodium, magnesium, calcium, potassium, among others) that are potentially transferred to human sweat and then to the skin (Carretero et al. 2010; Fernández-González et al. 2021). Despite the lower absorption capacity, compared to the remaining ones, seawater peloids may be able to provide ions to the skin, depending on their concentration in samples (Matike et al. 2011). This exchange of nutrients (macro and/or micro), that were adsorbed by clay minerals, during the maturation process ensure that cleansing occurs through toxin removal, bacteria, and unwelcome components by absorbing them, but can also transport harmful compounds (Tateo and Summa 2007; Matike et al. 2011).

The zeta potential indicates the degree of electrostatic repulsion between charged particles, revealing the kinetic stability of the colloidal suspension (such as clays in water) (Bastos and Rocha 2023). In line with Kadu et al. (2010), particles with a ZP value above − 30 or 30 mV are considered kinetically stable, which means that particles manage to repel each other (disaggregation and deflocculation). One the other hand, low ZP values suggest particles aggregation (and flocculation) due to Van der Waals attractive forces, as occurs in samples under study (Fig. 2). Clay minerals, such as smectite, are recognized for displaying a variable zeta potential according to suspension pH, ionic strength, type of ionic species existing and temperature (Saka and Güler 2006). The ZP values of samples tend to become more negative as the pH increases (Fig. 2). The growth of zeta potential at high pH levels is primarily attributed to the OH^-^ ions interactions with positively charged edges of clay minerals particles, which become neutral or even negatively charged due to hydroxyl ion adsorption (Vane and Zang 1997). Peloids for therapeutic and dermocosmetic applications must have an accurate zeta potential which have an influence on texture, plasticity, viscosity and adhesion to skin (rheological characteristics) (Khiari et al. 2019).

Furthermore, also there is no specific national or international legislation for the microbiological quality of pelotherapy products, however the European Comission endorses actual attention to microbiological purity of topical products to be used on mucous membranes. Baldovin et al. (2020) also stress the relevance of microbiological quality of products, if used by immunocompromised or elderly individuals, due to their physiological immunosenescence.

The quantification of culturable microorganisms, namely aerobic mesophilic bacteria, is an important indicator of the microbiological quality of pharmaceutical and cosmetic products (Carretero 2020b). Aerobic mesophilic bacteria are widespread in nature and are one of the most common descriptors of the microbial load in natural and manufactured products, being an unspecific indicator of microbiological contamination or pollution (Bashir and Lambert 2020; Madigan et al. 2021).

In accordance with ISO 17516:^2014^: Cosmetics - Microbiology – Microbiological limits, products specifically intended for children under three years of age, the eye area or the mucous membrane, should not exceed the limit ≤ 10^2^ CFU/g. Moreover, the regulations given by USP 1111 (2012) - Microbiological examination of nonsterile products: acceptance criteria for pharmaceutical preparations and substances for pharmaceutical use – are dependent on the route of administration. Both cutaneous use and transdermal patches have a recommended limit of ≤ 10^2^ CFU/g. Thermo-mineral water peloids and seawater peloids showed CFU/g above the recommended limits.

FIB is commonly used to assess the quality of beach and spa waters, as well as pharmaceutical and/or cosmetic products (Baldovin et al. 2020; Searcy et al. 2023). Overall, the results indicate that although the bacterial load, as expressed by the concentration of total mesophilic bacteria, tends to exceed the recommended limits, bacteria originate from natural environmental sources, considering that FIB is virtually absent. These results are in line with the characterization of Portuguese peloids reported by Bastos and Rocha (2023), where colony-forming units per gram range between 10^3^ − 10^5^ CFU/g.

Yeasts and moulds are aerobic fungi that can be found in natural products such as peloids, and their development is favoured by exposure to air. According to Lampropoulou et al. (2023) and Kataržytė et al. (2024), contact with pathogenic species elevates the risk of skin infections, as dermatitis or fungal infections (onychomycosis). The ISO 17516:^2014^ sets a recommended limit for the concentration of yeasts and moulds of ≤ 10³ CFU/g. In products specifically intended for children under three years of age, the eye area or the mucous membrane, the limit is ≤ 10^2^ CFU/g. Moreover, the regulations given by USP 61 (2022) - Microbiological examination of nonsterile products: microbial enumeration tests – and USP 62 (2022) - Microbiological examination of nonsterile products: tests for specified microorganisms – also recommend a general limit of ≤ 10^2^ CFU/g. Thermo-mineral water peloids have higher fungal content (10^2^ – 10^3^ CFU/g) than seawater peloids (around 10^1^ CFU/g). Beja-S, Bena-S and Monta-S comply with the ISO 17516:^2014^ and USP 61 and 62 recommended limits and are comparable with the values of 10^1^ CFU/g reported by Sánchez-Espejo et al. (2014) in a pharmaceutical formulation, commonly used in spa centres of southern European countries (Italy, Spain and Tunisia).

## Conclusions

The influence of thermo-mineral water and seawater is evident in the physico-chemical characteristics of peloids. On one hand, thermo-mineral water preserved the alkaline pH values of Beja-T, Bena-T and Monta-T (7.9–8.5), the EC values (0.7–0.9 mS/cm) and the OM content (2.6–4.6%), as well as stabilizing the ZP values throughout the maturation process. However, it enhanced the CEC of peloids (from 38.4 to 70.0 meq/100 in starting samples to 55.2–86.6 meq/100 g). On the other hand, seawater, although it also preserved the alkaline pH values of Beja-S, Bena-S and Monta-S (7.1–7.9) and ZP values, increased the OM (from 2.6 to 4.7% in starting samples to around 7%) and EC values (from 0.3 to 0.5 mS/cm in starting samples to around 70.0 mS/cm), however the CEC decreased. Moreover, samples exhibited concentrations of Pb, Co, Ni and V above the acceptable limits for cosmetic and pharmaceutical products. However, these samples may still be considered safe when supported by robust evidence demonstrating minimal systemic absorption and an acceptable risk-benefit balance. Therefore, further dermal bioaccessibility assessment and cutaneous uptakes are essential to establish whether these samples pose a genuine health concern or remain suitable for controlled therapeutic and dermocosmetic applications.

Furthermore, total mesophilic bacteria in seawater peloids slightly exceeds the limit recommended, the comparatively lower microbial load of seawater peloids, in relation to thermo-mineral water peloids, and the absence of FIB, make them preferable candidates for therapeutic applications. Thermo-mineral water peloids, present microbial loads exceeding recommended limits. Appropriate treatment to effectively reduce microorganisms without altering their rheology, mineral composition, or organic components (mild heat treatment, gamma irradiation, UV-C exposure, or high-pressure processing) would be required for therapeutic applications.

## Data Availability

The original contributions presented in the study are included in the article, further inquiries can be directed to the corresponding author.
